# Gene cloning of an important eukaryotic translation initiation factor family, *eIF2A* gene in halophytic *Leymus chinensis* (Trin.)

**DOI:** 10.1080/13102818.2014.948537

**Published:** 2014-10-21

**Authors:** Cheng-Wu Jin, Yan-Lin Sun, Soon-Kwan Hong

**Affiliations:** ^a^ College of Food Engineering, Ludong University, Yantai, China; ^b^ School of Life Sciences, Ludong University, Yantai, China; ^c^ College of Biomedical Science, Department of Bio-Health Technology, Kangwon National University, Chuncheon, Korea; ^d^ Institute of Bioscience and Biotechnology, Kangwon National University, Chuncheon, Korea

**Keywords:** *Leymus chinensis*, eukaryotic translation initiation factor, *eIF2A*, gene cloning

## Abstract

Eukaryotic initiation factors eIF2A and eIF2 both play important roles in the mRNA translation of protein synthesis, whereas the functions of eIF2A are usually overlooked, as both functions of binding methyionly-tRNA_i_ (Met-tRNA_i_) to 40S are similar under the same complementary factor and nucleotide requirements. Recently, the functions of eIF2A were reported to differ from those of eIF2 in manners when binding Met-tRNA_i_ to 40S. Given that eukaryotic initiation factor eIF2 has been well known, eIF2A was still deficient in understanding of its sequence, structure and functions. In this work, we collected a high salt-tolerant grass *Leymus chinensis* (Trin.) as the object of study, and cloned and sequenced the *eIF2A* gene from this species. Based on the DNA alignment and analysis of *eIF2A* gene sequences from other organisms, an effective primer set was newly designed. Using this primer set, a DNA fragment with length of about 500 bp was obtained, and we have submitted this sequencing result to NCBI GenBank database (accession number: KF279515). The Basic Local Alignment Search Tool (BLAST) result showed that our sequence is highly identical to eIF2A gene sequences that existed in NCBI GenBank database. This work would help to further understand the function of *eIF2A*, and provide more potential target genes for studying their functions in relation to stress tolerance mechanisms.

## Introduction

The process of translation initiation is the start of protein synthesis, and involves the binding of specific initiator methyionly-tRNA_i_ (Met-tRNA_i_) to the small ribosomal subunit, formation of ribosomal initiation complex and locating the initiation codon, and joining of the large ribosomal subunit to generate a translation-competent ribosome.[1] During these processes, more than 12 protein factors called translation initiation factors (eIFs) participate and play very important roles by interacting with ribosomal subunits.[2] Among these eIFs, the eukaryotic translation initiation factor 2 gene (*eIF2*), originally identified over 35 years ago, catalyses the formation of preinitiation complex in the process of protein synthesis.[3] As commonly known, eIF2 binds GTP and Met-tRNA_i_ to first form a ternary complex, and then transfers Met-tRNA_i_^(Met)^ to the 40S ribosomal subunit.[4] After forming the eIF2-GTP-Met-tRNA_i_ ternary complex, it is bound with the 40S ribosomal subunit to form the 43S preinitiation complex. During the process of the 43S preinitiation complex formation, the translation initiation factors eIF2A and other two factors including eIF1 and eIF3 are considered to participate and stimulate this process.[5] Although both eIF2 and eIF2A function in the methionyl-puromycin assays, the events that occur differed between them. eIF2 binds Met-tRNA_i_ to 40S subunits in a GTP-dependent manner, whereas eIF2A binds Met-tRNA_i_ to 40S subunits in a codon-dependent manner.[3] Given that the eIF2 and eIF2B factors among the initiation factor 2 family genes have been highly known, eIF2A factor is considered to function in a minor pathway or in the translation of a small number of specific mRNAs,[3] resulting in deficient understanding of its role. Thus, this work focuses on the cloning of eIF2A, which has been successfully done by us. We cloned the *eIF2A* gene from a high salt-tolerant grass *Leymus chinensis* (Trin.) to provide more sequence sources for further comparison of eIF2A among other organisms.

*L. chinensis*, a perennial rhizome grass in the Gramineae family, is widely distributed throughout northern China, Mongolia and Siberia,[6] having intrinsic potentials to thrive under environmental highly alkaline-sodic soil conditions (pH 8.5–11.5).[7] Due to its characteristics, *L. chinensis* has been used as a soil-binding plant to protect soil from desertification in northern China. According to the previous studies, salt-tolerant plant species usually have halophytic ancestors, so these grass species are considered as a potential source of halotolerance genes for glycophytic crop plants. In this work, we describe cDNA clone encoding the *L. chinensis eIF2A* gene (*LceIF2A*). The results from the current research would help to further understand the function of *eIF2A*, and would provide more potential target genes for studying their functions in relation to stress tolerance mechanisms.

## Materials and methods

### Plant materials

Mature seeds of China wild-type *L. chinensis* plants, LcWT07, were obtained from natural grassland in Songneng Plain, Siping, Jilin, China. These seeds have been optimized for this environment by many years of natural evolution. The mature seeds were dipped with water at 4 °C for 3 days, and those sinking to the bottom were selected and grown in pots containing clay/vermiculite (3/1, v/v). The cultures were kept at 25 °C in greenhouse conditions with a 16/8 h (day/night) photoperiod and a relative humidity between 45% and 70%. Seedlings were watered daily with Hoagland nutrient solution.[8]

### Sequence BLAST and primer design

Information for the *eIF2A* gene of several organisms was obtained from the NCBI database, including *Arabidopsis thaliana* (accession number: NM001036441); sorghum (*Sorghum bicolor*, accession number: XM002452372); Pooideae grass (*Brachypodium distachyon*, accession number: XM003575271); bread wheat (*Triticum aestivum*, accession number: GAJL01268064); honey bee (*Apis mellifera*, accession number: XM625140); *Homo sapiens* (accession number: AK298586); *Ostreococcus tauri* (accession number: XM003074869); *Plasmodium falciparum* (accession number: XM001348498); and Epizootic hematopoietic necrosis virus (accession number: AJ130965), and these eIF2A mRNA sequences were then compared with each other. Three sets of primers for amplification of *LceIF2A* were designed according to the regions with conserved sequences. The location and nucleotide sequences of the forward and reverse primers are shown in Table 1. The newly designed primers were named as the objective gene name (eIF2a), followed by ‘–’ + letter + number. Letter f or r represents the direction of primers, deriving from the initial of forward or reverse, respectively. The numbers followed by the letter are given according to the order, to discriminate each primer. Any forward primer could be matched with any reverse primer, such as eIF2a-f1/eIF2a-r1, eIF2a-f2/eIF2a-r1 and eIF2a-f3/eIF2a-r2.

**Table 1:** . The newly designed primer nucleotide sequences for this study.

Primer name	Nucleotide sequence
eIF2A-f1	5′-ATGRCATCKCCACCGYCG-3′
eIF2A-f2	5′-GGCTKCACKGTGTGGWMT-3′
eIF2A-f3	5′-ACCAACCARAGTTACTATGG-3′
eIF2A-r1	5′-TCAACTTTCATCAGCAGG-3′
eIF2A-r2	5′-TTTCCAWTCAGCCTGATACAG-3′
eIF2A-r3	5′-ATAGTAACTYTGGTTGGT-3′

### PCR amplification and sequencing

Genomic DNAs were extracted from fresh leaf tissues of wild-type and transgenic plant using sodium dodecyl sulphate (SDS) method.[9] polymerase chain reaction (PCR) reactions were carried out in a total volume of 20 μl with the following reaction mixture: 0.5 μmol/l of each primer, 10 ng DNA template, 200 μmol/l of each dATP, dCTP, dGTP and dTTP mix, 1.0 unit of Taq DNA polymerase (Promega, USA) and corresponding buffer. PCR amplification was performed with an initial denaturation at 95 °C for 5 min followed by 35 cycles of incubation at 95 °C for 30 sec, incubation at the corresponding annealing temperatures of each primer sets for 30 sec and extension at 72 °C for 30 sec, with a final extension step at 72 °C for 5 min. PCR products were analysed by 1% agarose gel electrophoresis. According to the combination of differently assembled primer sets, the PCR products varied in length. The obtained cDNA sequence and genomic sequence from the *LceIF2A* gene were then deposited at the NCBI database, and assigned an accession number: KF279515.

### Sequencing analysis

The assembled cDNA sequences and genomic DNA sequences were analysed by the software DNAMAN 6.0 version. Homologous sequences to the ones obtained by us were selected and nucleotide sequence comparisons were performed through the Basic Local Alignment Search Tool (BLAST) network services against databases (http://www.ncbi.nlm.nih.gov/). The multiple sequence alignment of *eIF2A* genes among different species were also performed using the DNAMAN version 6.0 software.

## Results and discussion

**Figure 1.**
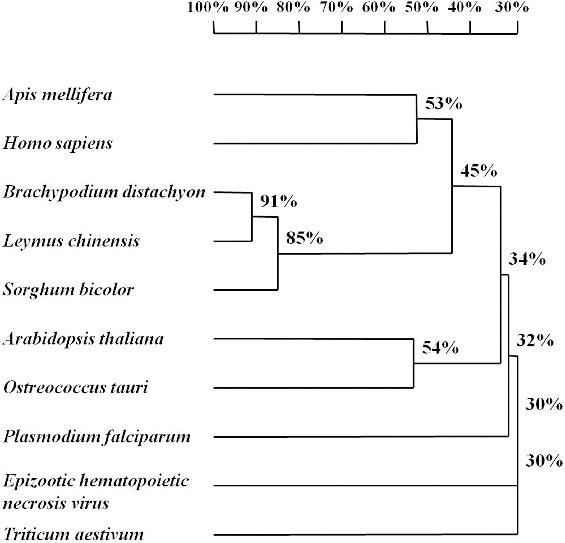
Phylogenetic tree constructed using eIF2A sequences of 9 organisms and *Leymus chinensis* (Trin.) by software DNAMAN 6.0 version.

**Figure 2.**
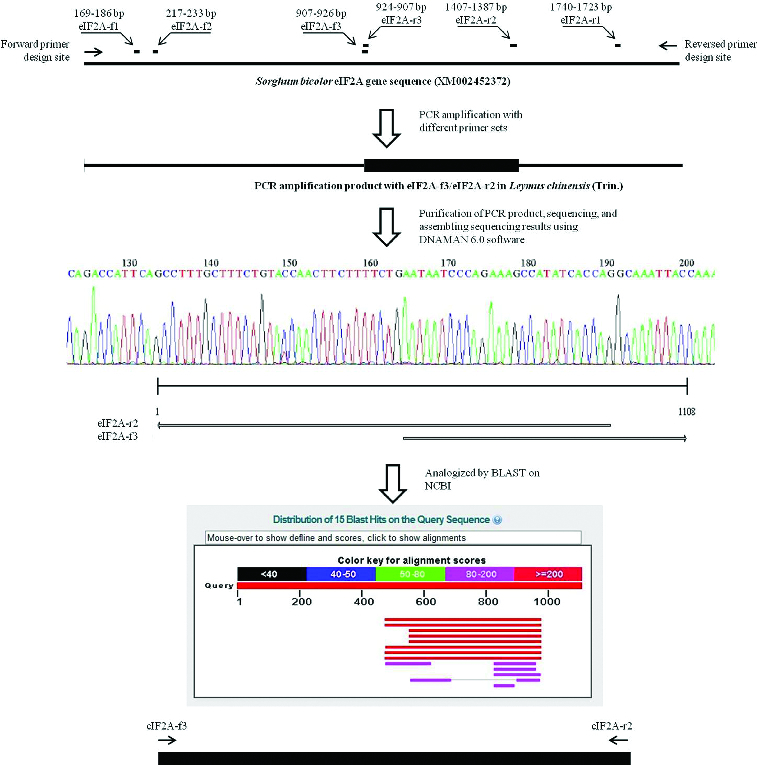
Process of gene cloning of the positive gene, *LceIF2A* and subsequent sequence analysis.

Systematic BLAST searches in the National Center for Biotechnology Information (NCBI, http://www.ncbi.nlm.nih.gov/) led to identification of 9 *eIF2A* genes from: *Arabidopsis thaliana* (accession number: NM001036441); sorghum (*Sorghum bicolor*, accession number: XM002452372); Pooideae grass (*Brachypodium distachyon*, accession number: XM003575271); bread wheat (*Triticum aestivum*, accession number: GAJL01268064); honey bee (*Apis mellifera*, accession number: XM625140); *Homo sapiens* (accession number: AK298586); *Ostreococcus tauri* (accession number: XM003074869); *Plasmodium falciparum* (accession number: XM001348498); and Epizootic hematopoietic necrosis virus (accession number: AJ130965). In order to compare the similarities among them and find the conserved sequence regions for primer design, we constructed a sequence alignment and a phylogenetic tree based on the nucleotide homology with our *LceIF2A* sequence (Figure 1). Seen from the phylogenetic tree, the *LceIF2A* sequence showed 91% and 85% similarity to the sequences of *Brachypodium distachyon* and *Sorghum bicolor*, respectively (Figure 1). Due to sequence length and similarity, we retrieved the *Sorghum bicolor eIF2A* gene sequence (XM002452372) and used it as the template for the *eIF2A* primer design (Figure 2). The designed primers for amplification of *LceIF2A* according to the conserved sequences are shown in Table 1 and their exact location in the used sequences is presented in Figure 2. Using the eIF2A-f3/eIF2A-r2 primer set, a DNA fragment with a length of about 500 bp was obtained, and submitted as a sequencing result to the NCBI GenBank database (accession number: KF279515).

Following the sequencing and assembly of our sequencing data, we performed a search with it using NCBI BLAST. The BLAST result showed that our sequence was most similar to transcribed RNA sequences from *Triticum aestivum* and *Triticum turgidum* (accession number: GAJL01268064), and had 99% similarity to *Brachypodium distachyon* eukaryotic translation initiation factor 2A-like (LOC100841968) mRNA sequence (accession number: XM003575271). Thus, the assembled sequence from *L. chinensis* was recognized as *eIF2A* gene.

**Figure 3.**
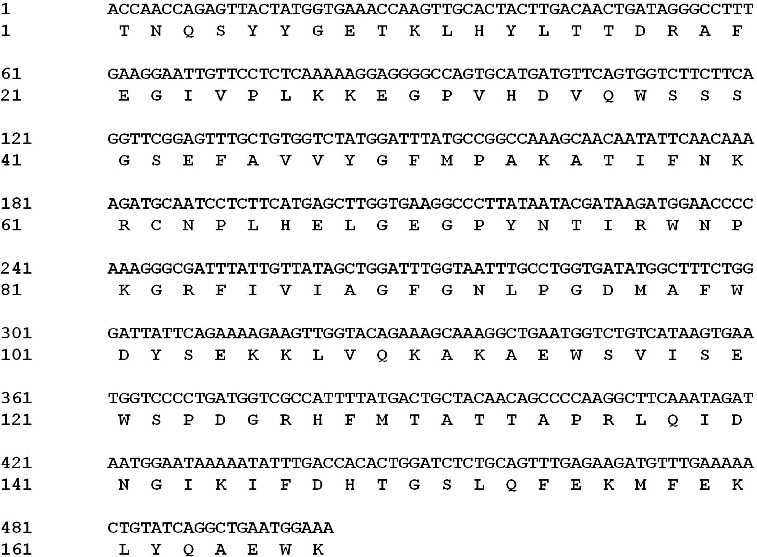
Amino acid sequence translated by *LceIF2A* gene sequence (accession number: KF279515).

The nucleotide sequence from the *LceIF2A* gene was translated into an amino acid sequence (167 amino acids) using software DNAMAN 6.0 version and shown in Figure 3. The protein sequence showed high similarity to EIF2A, further supporting the results and conclusion that it belonged to *eIF2A* gene sequence.

## Conclusions

This work provided a simple method for cloning of positive genes and identification in *L. chinensis*. This result would help to further understand the function of EIF2A during translation initiation or in relation to stress tolerance.
